# Factors associated with treatment outcome of MDR/RR-TB patients treated with shorter injectable based regimen in West Java Indonesia

**DOI:** 10.1371/journal.pone.0263304

**Published:** 2022-01-28

**Authors:** Arto Yuwono Soeroto, Raden Desy Nurhayati, Aga Purwiga, Bony Wiem Lestari, Chica Pratiwi, Prayudi Santoso, Iceu Dimas Kulsum, Hendarsyah Suryadinata, Ferdy Ferdian

**Affiliations:** 1 Faculty of Medicine, Division of Respirology and Critical Care Medicine, Department of Internal Medicine, Hasan Sadikin General Hospital, Universitas Padjadjaran, Bandung, West Java, Indonesia; 2 Faculty of Medicine, Department of Internal Medicine, Hasan Sadikin Hospital, Universitas Padjadjaran, Bandung, West Java, Indonesia; 3 Department of Internal Medicine, Rotinsulu Pulmonary Hospital, Bandung, West Java, Indonesia; 4 Faculty of MedicineDepartment of Public Health, Universitas Padjadjaran, Bandung, West Java, Indonesia; 5 Faculty of Medicine, TB-HIV Research Center, Universitas Padjadjaran, Bandung, West Java, Indonesia; 6 Department of Internal Medicine, Cimacan Hospital, Cianjur, West Java, Indonesia; The University of Georgia, UNITED STATES

## Abstract

**Background and aims:**

Multi drug or rifampicin resistant tuberculosis (MDR/RR-TB) is a major burden to TB prevention and eradication globally. Since 2016, WHO guidelines have included options for treating MDR/RR-TB with a standard regimen of 9 to 11 months duration (the ’shorter regimen’) rather than an individual regimen of at least 20 months. This regimen has been introduced in Indonesia since September 2017. Therefore, we aimed to determine the success rate and factors associated with the treatment outcome of shorter injectable based regimen in West Java province, Indonesia.

**Methods:**

This was a retrospective cohort study of MDR/RR-TB patients aged over 18 years old who received the shorter injectable based regimen between September 2017 and December 2020. We defined successful outcomes as the combined proportion of patients who were cured or had complete treatment. While, unsuccessful outcomes were defined as the combined proportion of patients who died from any causes, failure, and loss to follow-up (LTFU).

**Results:**

A total of 315 patients were included in this study. The success rate was 64.5%. Multivariate analysis showed male gender (aRR = 1.18, 95% CI 1.04 to 1.34) increased the chance of successful outcome, while malnutrition (aRR = 0.78, 95% CI 0.68 to 0.89), history of previous TB treatment (aRR = 0.80%CI 0.68 to 0.94), and time of culture conversion >2 months (aRR = 0.72 (95% CI 0.59 to 0.87) decreased the chance of successful outcome.

**Conclusion:**

History of previous TB treatment, time of culture conversion >2 months, and malnutrition were independent factors that decrease the chance for success rate, while male gender increase the likelihood for success rate of patients treated by the shorter injectable based regimen.

## Introduction

Multidrug-resistant tuberculosis (MDR/RR-TB), defined as Mycobacterium *tuberculosis* infection with resistance to rifampicin or both isoniazid and rifampicin, has become a significant problem on TB eradication globally [1]. Based on World Health Organization (WHO) global report in 2019, there were around 465,000 cases of MDR/RR-TB globally, with a proportion of 3.3% of new TB patients and 18% of patients with previous TB treatment [2]. In 2017, there were 558,000 cases of MDR/RR-TB, and about 8.5% of MDR-TB cases become extensive drug-resistant TB (XDR-TB) [3]. In Southeast Asia, TB incidence has reached 4,340,000 cases, of which 171,000 are MDR/RR-TB cases [2]. According to WHO, Indonesia is one of the countries with a high TB burden. High burden TB is determined based on three indicators: TB, TB / HIV (Human Immunodeficiency Virus), and MDR-TB. The thirty countries with the highest TB burden account for nearly 86% of new TB cases globally, and eight of these contributed two-thirds of the total, with Indonesia ranks third after India and China [1].

Multidrug-resistant TB is one of the main barriers to TB prevention and eradication globally. The cure rate for drug-resistant TB was much lower than for drug-sensitive TB in patients on complete treatment. In addition, MDR/RR-TB treatment is also more expensive, and many patients experience side effects during treatment [4]. The World Health Organization recommends identifying the factors that influence the duration and outcome of treatment as a research priority, particularly in high-burden countries [5]. Previously, MDR/RR-TB required a minimum treatment period of more than 20 months. Prolonged treatment is expensive, poorly tolerated, and associated with high rates of adverse events [6,7]. The success rate of MDR/RR-TB treatment is still low, only around 57% globally [2]. Thus, the need for a regimen that is less time-consuming but effective is essential. Since 2016, WHO guidelines have included options for treating MDR/RR-TB with a standard regimen of 9 to 11 months duration (the ’shorter regimen’) rather than a longer individual regimen of at least 20 months [7]. The shorter regimen TB was based on observational studies in several countries that have implemented it before, including Bangladesh, Benin, Burkina Faso, Burundi, Cameroon, Central Africa, Congo, Niger, Swaziland, and Uzbekistan. The results showed that the success rate of the short-term treatment regimen was higher than that longer regimen [8]. Many studies have shown a varying success rate of shorter regimen treatment, between 44.3%– 83.7% [9–11]. Given this varied range of success rate, our study aimed to identify the success rate and factors associated with the outcome of shorter injectable based regimens in MDR-TB patients in West Java province, Indonesia.

## Materials and methods

### Setting

This was a retrospective cohort study of MDR/RR-TB patients in Hasan Sadikin General Hospital, Bandung, Indonesia. This hospital is a tertiary referral hospital and as one of drug-resistant TB treatment centers, it serves approximately 40% of total MDR/RR-TB patients in West Java province. According to the National TB Programme (NTP) guideline, presumptive TB cases who were tested by Xpert MTB/RIF assay and had rifampicin-resistant result were classified as MDR/RR-TB patients. These patients, then, completed a series of pre-treatment examination and treated by certain MDR/RR-TB regimens according to the clinical expert team. MDR/RR-TB treatment is provided and organized by the NTP.

### Subjects selection

We recruited all MDR/RR-TB patients aged over 18 years old who received a shorter regimen between September 2017 and December 2020. Data were collected from medical record. Rifampicin resistance was confirmed by a phenotypic test (drug susceptibility testing) or genotypic test (Xpert MTB/RIF assay). All patients met WHO criteria for shorter regimen, including pulmonary tuberculosis, no exposure for ≥1 month to second-line drugs included in the shorter regimen, no pregnancy, no history of confirmed resistance to all drugs included in shorter regimens except isoniazid, and no intolerance or risk of toxicity to medicines included in the shorter regimen. Patients with HIV, known resistance to second-line drugs, still on treatment, transferred to other hospitals, and refuse medication were excluded from the study. Patients with no sputum culture data in the first one to three months of treatment, despite the availability of treatment outcome, were also excluded after screening process. We have analyzed the exclusion, which result in no selection bias, as the analysis was attached as S1 Table. This study had received approval from the Health Research Ethics Committee of Hasan Sadikin General Hospital number LB.02.01/X.6.5/20/2021.

### Shorter regimen definition

Shorter regimens defined as standardized regimens, consist of two phases: intensive and continuation phase. In our study, we used the shorter injectable based regimen. The intensive phase includes 4 to 6 months of kanamycin, moxifloxacin, ethionamide, high-dose isoniazid, clofazimine, pyrazinamide, ethambutol, followed by a continuation phase with five months of moxifloxacin, clofazimine, pyrazinamide, and ethambutol. The following substitutions were allowed: amikacin or capreomycin instead of kanamycin, levofloxacin instead of moxifloxacin.

### Patients follow-up and outcome definition

Patient’s information including body mass index (BMI), anemia (Hb < 12g/dl for women and Hb <13 g/dl for men), diabetes mellitus type 2 (DM type 2), chronic kidney diseases, rapid molecular test using Xpert MTB/RIF assay, cavities on chest X-ray (CXR), past history of TB treatment were collected before shorter regimen given. Sputum culture is taken as a follow-up assessment, which considered to have converted when three consecutive cultures are found to be negative in span of 30 days [12]. Assessment of culture conversion using liquid culture was collected monthly during follow-up in the MDR-TB clinic of Hasan Sadikin General Hospital by the physician in charge. The successful outcome was defined as the combined proportion of cured patients (complete treatment with negative culture results for at least three consecutives with an examination interval of at least 30 days during follow-up) and had complete treatment. The unsuccessful outcome was defined as the combined proportion of patients who died from any causes, failure (withdrawal because of severe drug’s side effect, additional acquired resistance to quinolone or second-line injectable agents, no sputum smear conversion after six months or at the end of treatment, bacteriological reversion in continuation phase after conversion to negative) and loss to follow-up (LTFU, defined as patients whose treatment was interrupted for two consecutive months or more).

### Data analysis

Continuous variables with normal distribution were described as mean±standard deviation (SD). Non-normal distribution data were presented as the median and interquartile range (IQR). Categorical variables were presented in percentages (%). Chi-square or Fisher exact test was used to compare categorical variables. Variables with a p-value <0.25 and missing data less than 10% were included in multivariate analysis [13]. The outcome was reported using adjusted relative risk (aRR) and 95% CIs using log-binomial regression. Variables included in multivariate analysis were classified into two values. We considered a statistical significance when the p-value ≤ 0.05. All analysis was performed using STATA version 16 for Windows.

## Results

### Baseline characteristics

**Fig 1:**
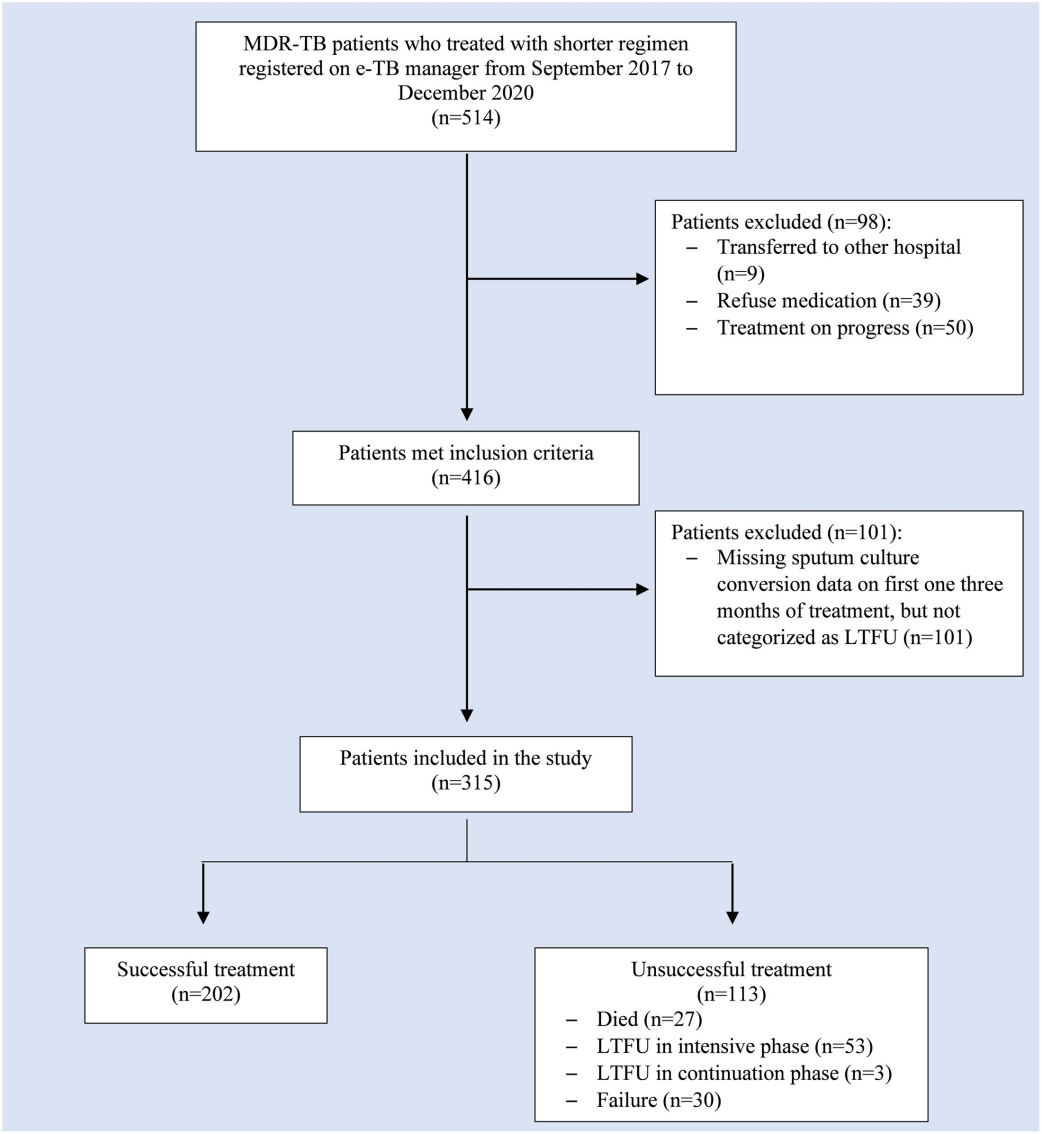
Flow chart of study participants. MDR/RR-TB: Multi-drug and rifampicin resistant TB. LTFU: Loss to Follow Up.

There were 1193 MDR/RR-TB patients registered in Hasan Sadikin General Hospital e-TB Manager between September 2017 and December 2020. Of 1193 MDR/RR-TB patients, 514 adult cases were treated with the shorter regimen. At first admission, the patient’s consent for the use of the shorter regimen was obtained. Thirty-nine patients refused shorter regimen treatment. A total of 50 patients were excluded from the study since they were still on treatment at the time of recruitment, and nine patients were transferred to other hospital. After screening process, 101 patients were excluded due to no sputum culture data in the first one to three months of treatment, despite the availability of treatment outcome. A total of 315 patients were included in this study. Patients’ enrollment is shown in Fig 1.

Most patients were aged ≤45 years old (68.2%). The male proportion was slightly higher than the female in the unsuccessful and successful groups (50.4% vs. 59.4%). Most patients’ sputum culture converted to negative in less than two months (62.5%). There were 30 patients with no sputum data conversion in the unsuccessful group due to LTFU in the first three months of treatment. Regarding history of previous TB treatment, 47.3% were relapse cases, followed by failure cases (24.7%), LTFU (14.0%) and new TB patient (14.0%). Overall, the proportion of successful outcomes was higher than unsuccessful (64.5% vs. 35.5%). In unsuccessful group, there were 27 (23.9%) patients died, 56 (49.5%) LTFU, and 30 (26.6%) failed to shorter regimen.

### Univariate analysis

Univariate analysis showed an underweight reduce the chance of successful outcome compared to normal BMI with RR = 0.77 (95% CI 0.66 to 0.91, p = 0.003). In addition, patients with anemia had a lower chance of successful outcomes than those without anemia with RR = 0.90 (95% CI, 0.70–0.99, p = 0.04). Sputum culture conversion for more than two months also reduces the chance of successful outcome with RR = 0.71 (95% CI, 0.58 to 0.87, p = 0.001). Overall, there was no significant association between previous TB treatment (relapse, failure, LTFU) and successful outcomes. However, patients with a history of failure on previous TB treatment had a lower chance of successful outcome than patients with new TB patients with RR = 0.73 (95%CI 0.58 to 0.87, p = 0.02). The baseline characteristics and univariate analysis are shown in Table 1.

**Table 1:** Baseline characteristics and univariate analysis.

	Unsuccessful (n = 113)	Successful (n = 202)	*p-value*	*Crude RR* (95%CI)
**Age (years)**			0.59	
**Median (Range)**	40 (24)	18 (17)		
≤ 45	75 (66.4)	140 (69.3)		1 (Reference)
>45	38 (33.6)	62 (30.7)		0.95 (0.79–1.14)
**Gender**			0.12	
Female	56 (49.6)	82 (40.6)		1 (Reference)
Male	57 (50.4)	120 (59.4)		1.14 (0.96–1.36)
**BMI (kg/m** ^**2**^ **)**				
**Median (IQR)**	17.52 (3.30)	17.79 (3.98)		
<18.5	80 (70.8)	119 (58.9)	**0.003** *	0.77 (0.66–0.91)
18.5–22.9	19 (16.8)	63 (31.2)	-	1 (Reference)
23–24.9	7 (6.2)	10 (5.0)	0.20	0.76 (0.50–1.15)
25–29.9	6 (5.3)	10 (5.0)	0.30	0.81 (0.54–1.21)
≥30	1 (0.9)	-	-	-
**Anemia**			**0.04** *	
No	55 (47.8)	122 (60.4)		1 (Reference)
Yes	58 (51.3)	80 (39.6)		0.90 (0.70–0.99)
**DM type 2**			0.64	
No	86 (76.1)	149 (73.8)		1 (Reference)
Yes	27 (23.9)	53 (26.2)		1.04 (0.86–1.25)
**CKD**			0.55	
No	111 (98.2)	200 (99.0)		1 (Reference)
Yes	2 (1.8)	2 (1.0)		0.77 (0.29–2.07)
**Previous TB treatment**				
New TB patients	11 (9.7)	33 (16.3)	-	1 (Reference)
Relapse	51 (45.1)	98 (45.8)	0.21	0.87 (0.71–1.07)
Failure	35 (31.0)	43 (21.3)	**0.02** *	0.73 (0.56–0.95)
*Loss to follow up*	16 (14.2)	28 (13.9)	0.23	0.84 (0.64–1.12)
**Time of Culture Conversion**	**n = 83**	**n = 202**		
≤ 2 months	44 (53.1)	153 (75.7)	**-**	1 (Reference)
> 2 months	39 (46.9)	49 (24.3	**0.001** *	0.71 (0.58–0.87)
**Gene Xpert**	**n = 101**	**n = 188**		
Very low	4 (4.0)	5 (2.7)	-	1 (Reference)
Low	18 (17.8)	44 (23.4)	0.42	1.27 (0.69–2.34)
Medium	53 (52.5)	91 (48.4)	0.67	1.13 (0.62–2.06)
High	26 (25.7)	48 (25.5)	0.61	1.16 (0.63–2.14)
**Cavity on CXR**	**n = 93**	**n = 156**	0.38	
No	46 (49.5)	86 (55.1)		1 (Reference)
Yes	47 (50.5)	70 (44.9)		0.91 (0.75–1.11)

The dependent outcome for Crude RR: Successful outcome.

BMI: Body Mass Index; DM: Diabetes Mellitus; CKD: Chronic Kidney Disease; TB: Tuberculosis; CXR: Chest x-ray; RR: Relative Risk; CI: Confidence Interval. Categorical variables were presented as number (%).

* Statistically significant (p<0.05).

### Multivariate analysis

In log-binomial regression analysis, we selected five variables with p<0.25, including gender, body mass index, anemia, history of previous TB treatment, and time of culture conversion. The final model showed male gender (aRR = 1.18, 95%CI 1.04 to 1.34) increase the likelihood for successful outcome, while malnutrition (aRR = 0.78, 95% CI 0.68 to 0.89), history of previous TB treatment (aRR = 0.80, 95%CI 0.68 to 0.94), and time of culture conversion >2 months (aRR = 0.72 (95% CI 0.59 to 0.87) decrease the likelihood for successful outcome. Multivariate analysis is shown in Table 2.

**Table 2:** Multivariate analysis.

Variables	Initial model		Final Model	
	aRR (95% CI)	p-value	aRR (95% CI)	p-value
Male	1.17 (1.03 to 1.33)	0.01	1.18 (1.04 to 1.34)	0.009
Malnutrition^a^	0.80 (0.69 to 0.97)	0.002	0.78 (0.68 to 0.89)	<0.0001
Anemia^b^	0.90 (0.77 to 1.07)	0.25		
History of Previous TB Treatment^c^	0.76 (0.63 to 0.91)	0.004	0.80 (0.68 to 0.94)	0.007
Time of **Culture Conversion** (>2 months)^d^	0.73 (0.60 to 0.89)	0.002	0.72 (0.59 to 0.87)	0.001

Dependent variables: Successful outcome.

BMI: Body Mass Index; TB: Tuberculosis; RR: Relative Risk.

^a^) underweight, overweight, and obese compared to normal;

^b^) anemia compared to non-anemia;

^c^) relapse failure, loss to follow-up compared to new TB patients;

^d^) culture conversion >2 months compared to ≤2 months.

* Statistically significant (p<0.05).

## Discussion

The result of this study showed that male gender was independent factor that increase the chance of successful outcome, while history of previous TB treatment, time of sputum culture conversion >2 months, and malnutrition, particularly underweight, decrease the chance for successful outcomes for MDR/RR-TB patients treated with the shorter regimen. In previous studies, several factors have been identified as predictors of poor MDR/RR-TB treatment outcome, including older age, male, history of resistance to ofloxacin and other second-line drugs, cavities on chest x-ray, delayed sputum culture conversion, positive AFB smear at diagnosis, and presence of comorbid (HIV, DM type 2, malnutrition) [14–18]. Many attempts have been made to increase the success rate of MDR/RR-TB treatment. In 2016, WHO introduced the shorter regimen as a new MDR/RR-TB treatment to reduce the LTFU rate, reducing the unsuccessful treatment outcome. A previous study investigating longer regimen in MDR/RR-TB patients in Indonesia showed a 50% successful outcome rate. Age, gender, BMI, previous TB treatment, time of culture conversion, acid-fast bacilli (AFB) smear, HIV, chronic kidney disease (CKD), and cavities on chest radiograph were the predictors of the longer regimen outcome [19]. In this paper, we discovered that the shorter regimen for MDR/RR-TB treatment appears to have a better outcome with success rate of 65%, which was higher than the longer regimen from the same setting [19]. However, the longer regimen can be used in most MDR/RR-TB patients, whereas the shorter regimen can only be used in certain patients [20].

Contrary to a prior study, we found that male gender were associated with a successful treatment outcome. A previous study showed males were likely to develop unsuccessful MDR/RR-TB treatment OR = 1.40, 95% CI: 1.28–1.52 compared to females. The factor that may play an important role is the behavior of drinking alcohol and drug abuse [21]. This is in accordance with The European Monitoring Centre for Drugs and Drug Addiction report that stated these behaviors are more common in men [22]. Males were also associated with poor outcomes treatment due to delayed and non-compliance treatment [23]. Nonetheless, alcoholism and drug abuse are not common in our country. The different cultures may partially explain the different results.

A study conducted by Agustina et al. on 223 adult MDR/RR-TB patients treated with the shorter regimen in other Indonesia Tertiary Teaching Hospital showed a success rate of 46.6%. Patients older than 45 years old were likely to have a low success rate [24,25]. Age seems to play an important role in MDR/RR-TB treatment. Several studies from China and Ethiopia also shown that patients aged over 45 years old have a lower treatment success rate [17,26] Older age were associated with missed diagnosis, delayed treatment, and age-related diseases leading to advanced disease [21]. On the other hand, we found that older age was not associated with treatment outcomes. We did not investigate comorbidities that usually accompany older age, which may affect the successful treatment outcomes.

Patients with chronic renal failure tend to be more challenging to treat because they experience higher drug side effects and a progressive decline in kidney function [27,28]. In our study, the prevalence of CKD was very low and accounted for only 1–2% of cases in both groups. Thus, the exact relationship cannot be concluded. Diabetes mellitus increases the failure of TB treatment due to immune system disorders, kidney function disorders, the risk of drug toxicity, including the risk of liver toxicity [29,30]. Increased blood sugar exacerbates the clinical manifestations of TB, and conversely, TB can worsen blood sugar control. Complications in diabetes can potentially cause side effects of antituberculosis drugs, significantly impaired kidney function, and peripheral neuropathy [31]. A previous study also showed uncontrolled DM altering the immune response to M. *tuberculosis* [32]. However, HbA1c, which reflect diabetic control status, was not routinely measured.

Positive AFB smear at diagnosis is a strong predictor of delayed culture conversion time leading to poor MDR/RR-TB treatment outcomes [18,25,33]. Presence of cavities on CXR has been associated with a higher degree sputum smear positivity [34]. Patients with cavity lesions on chest x-ray had a lower likelihood of successful treatment [8,35]. Bilateral cavities are associated with prolonged culture conversion and failure of TB treatment. This is due to the presence of damaged lung parenchyma, which restricts drug penetration into the tissue [36,37]. A study conducted by Kurbatova et al. showed cavities on chest x-ray were associated with failure treatment with RR = 1.73 (95% CI 1.07–2.80) [25]. The morphology of cavities is heterogeneous. Most cavities are often located in the apical or posterior lobes. Thicker cavity wall was associated with higher M. *tuberculosis* concentration [38]. In this study, the characteristics of the cavity could not be described.

Anemia is a hematological disorder that is often found in TB patients. The degree of anemia in TB patients is generally mild and will improve with antituberculosis drug treatment [39]. MDR-TB patients experience lower serum iron and total iron-binding capacity (TIBC) levels compared to non-TB patients. Iron plays an essential role in any aspect of metabolic mechanism, including immune system modulation. Mycobacterial infection may induce inflammatory anemia by producing cytokines: interferon-γ, TNF-α, IL-1, and IL-10, leading to iron sequestration by macrophages and erythropoietin suppression [40]. Anemia also affects the culture conversion of MDR-TB patients, but the mechanism is not clear yet [41]. A study conducted by Irbah et al. showed anemia delays culture conversion in MDR-TB patients but is not associated with the type and severity of anemia [41]. Presence of anemia at baseline was associated with three times higher delayed sputum smear conversion with RR = 3.05 (95% CI 1.11 to 8.40) [42].

Weight loss is one of the main features in TB and MDR-TB patients [43]. Many developing countries are facing malnutrition problem, which give great impact on TB treatment. Both appear to have a reciprocal relationship. Undernourished TB patients were more likely to experience severe disease, treatment side effects, slow recovery rate, and death than patients with normal BMI [44]. Malnutrition, particularly underweight, disrupts the immune system against M. *tuberculosis* and is associated with a higher mortality rate of 1.9 times than MDR-TB patients with normal nutritional status [45]. Malnutrition may also result in secondary immunodeficiency that increases the risk of other infections [44]. Several studies showed that undernutrition is one of the contributing factors for poor outcomes in MDR-TB treatment [45–47]. Several studies also showed weight gain during treatment as an indicator of successful outcome [48,49]. Hence, nutrition intervention is an essential part of comprehensive MDR-TB treatment.

A meta-analysis conducted by Eshetie et al. showed that previous TB treatment was the most determining predictor for MDR-TB infection. Patients with previous TB treatment were 8.1 times more likely to develop MDR-TB [50]. Drug resistance usually arises from exposure to a single drug that inhibits susceptible bacilli but not pre-existing drug-resistant bacilli [51]. Previous TB treatment also affects culture conversion. A study conducted by Kurbatova et al. showed previous relapse or LTFU decrease chance of culture conversion in MDR-TB patients with Hazard Ratio = 0.69 (95% CI 0.54–0.88) compared to no previous treatment [52]. Sputum culture conversion within two months of treatment is a strong predictor of treatment success [53]. A previous study showed that patients with two months sputum culture conversion were more likely to exhibit successful outcomes with adjusted OR = 2.88 (95% CI: 1.11–7.45) [54]. Evidences suggested that sputum culture conversion in first two months may be an early predictor of higher treatment success rate and may shorten the treatment duration [55,56].

Interestingly, LTFU accounted for 49.5% of cases in an unsuccessful outcome. Many factors may be associated with successful outcomes. Non-compliance with treatment represents a complex view of patients’ personal and attitudes and health service capability, especially in Indonesia, where the quality of health services is not evenly distributed. Hasan Sadikin General Hospital is the tertiary referral hospital for MDR/RR-TB treatment in West Java. Many patients come from outside the city, which may require additional indirect health costs such as transportation cost, accommodation cost, etc. Patients who lived in rural areas also tend to get delayed diagnosis and treatment [57]. Consequently, the burden of MDR/RR-TB that we face may be much more significant than reported. Poverty, lack of access to healthcare, low education, and absence of a supporting system (e.g., family) may delay patients from follow-up treatment, leading to LTFU. Self-denial due to stigma experienced in society can also result in motivation loss. Hence, comprehensive MDR/RR-TB management involves a multidisciplinary approach is essential.

Our study is a retrospective cohort study, which thought to be the appropriate study design to assess risk factors. This research was conducted in Indonesia, one of the highest MDR-TB burden countries in the world, and large sample was involved, thus we hope to describe the overview of risk factors of TB treatment outcome properly.

### Study limitations

Some limitations should be noted. Since the data were taken retrospectively, there were missing or incomplete data, hence several variables that may affect treatment outcomes could not be identified and few subjects of study were unable to be assessed. As a part of management of missing value, we have analyzed the exclusion of 101 patients that met inclusion criteria, due to no sputum culture data in the first one to three months of treatment despite the availability of treatment outcome, and the result showed no significant variables difference, as shown in S1 Table. Therefore, this exclusion did not result to selection bias. Due to limited resources, the data for the 12 months post treatment follow-up were incomplete, thus we did not assess further.

## Conclusions

History of previous TB treatment, time of culture conversion >2 months, and malnutrition were independent factors that decreased the chance for success rate, while male gender increased the likelihood for success rate of patients treated by the shorter injectable based regimen. To achieve higher success rate, these population should be monitored carefully. Multidisciplinary approach is needed on treating TB, including nutritional intervention and management of concomitant diseases, in hope to increase the chance of successful outcome.

## Supporting information

S1 Table Analysis of excluded patients. The dependent outcome for Crude RR: successful outcome. BMI: Body Mass Index; DM: Diabetes Mellitus; CKD: Chronic Kidney Disease; TB: Tuberculosis; CXR: Chest x-ray; RR: Relative Risk; CI: Confidence Interval. Categorical variables were presented as number (%) * Statistically significant (p<0.05).
(DOCX)

S1 File (ODS)

S2 File (PDF)
