# Supplementary material for: Factors associated with treatment outcome of MDR/RR-TB patients treated with shorter injectable based regimen in West Java Indonesia
Source: PLoS One. 2022 Jan 28;17(1):e0263304. doi: 10.1371/journal.pone.0263304 (PMC8797248; doi:10.1371/journal.pone.0263304)
Supplement: S1 Table — The dependent outcome for Crude RR: successful outcome. BMI: Body Mass Index; DM: Diabetes Mellitus; CKD: Chronic Kidney Disease; TB: Tuberculosis; CXR: Chest x-ray; RR: Relative Risk; CI: Confidence Interval. Categorical variables were presented as number (%) * Statistically significant (p<0.05). (DOCX) [file pone.0263304.s001.docx]

|  | **N=315** | | | ***N=101*** | | |
| --- | --- | --- | --- | --- | --- | --- |
|  | **Unsuccessful**  **(n = 113)** | **Successful**  **(n=202)** | ***p-value*** | **Unsuccessful**  **(n = 53)** | **Successful**  **(n=48)** | ***p-value*** |
| **Age (years)**  **Median (Range)**  ≤ 45  >45 | 40 (24)  75 (66.4)  38 (33.6) | 18 (17)  140 (69.3)  62 (30.7) | 0.59 | 30 (56.6)  23 (43.4) | 25 (52.1)  23 (47.9) | 0.64 |
| **Gender**  Female  Male | 56 (49.6)  57 (50.4) | 82 (40.6)  120 (59.4) | 0.12 | 22 (41.5)  31 (58.5) | 23 (47.9)  25 (52.1) | 0.51 |
| **BMI (kg/m^2^)**  **Median (IQR)**  <18.5  18.5-22.9  23-24.9  25-29.9  ≥30 | 17.52 (3.30)  80 (70.8)  19 (16.8)  7 (6.2)  6 (5.3)  1 (0.9) | 17.79 (3.98)  119 (58.9)  63 (31.2)  10 (5.0)  10 (5.0) | **0.003***  -  0.20  0.30  - | 32 (59.2)  15 (28.5)  4 (8.2)  2 (4.1)  - | 38 (79.1)  8 (16.3)  2 (4.6)  -  - | 0.64 |
| **Anemia**  No  Yes | 55 (47.8)  58 (51.3) | 122 (60.4)  80 (39.6) | **0.04*** | 25 (47.9)  28 (52.1) | 23 (47.6)  25 (52.4) | 0.97 |
| **DM type 2**  No  Yes | 86 (76.1)  27 (23.9) | 149 (73.8)  53 (26.2) | 0.64 | 37 (69.2)  16 (30.8) | 37 (77.1)  11 (22.9) | 0.47 |
| **CKD**  No  Yes | 111 (98.2)  2 (1.8) | 200 (99.0)  2 (1.0) | 0.55 | 47 (89.6)  6 (10.4) | 45 (92.9)  3 (7.1) | 0.58 |
| **Previous TB treatment**  New TB patients  Relapse  Failure  *Loss to follow up* | 11 (9.7)  51 (45.1)  35 (31.0)  16 (14.2) | 33 (16.3)  98 (45.8)  43 (21.3)  28 (13.9) | -  0.21  **0.02***  0.23 | 5 (9.4)  23 (43.4)  14 (26.4)  11 (20.7 | 3 (6.3)  30 (62.5)  8 (16.7)  7 (14.6) | 0.29 |
| **Time of Culture Conversion**  ≤ 2 months  > 2 months | **n = 83**  44 (53.1)  39 (46.9) | **n = 202**  153 (75.7)  49 (24.3 | **-**  **0.001*** |  |  |  |
| **Gene Xpert**  Very low  Low  Medium  High | **n=101**  4 (4.0)  18 (17.8)  53 (52.5)  26 (25.7) | **n=188**  5 (2.7)  44 (23.4)  91 (48.4)  48 (25.5) | -  0.42  0.67  0.61 | 5 (9.5)  11 (21.4)  28 (52.4)  9 (16.7) | 4 (8.8)  8 (17.6)  23 (47.1)  13 (26.5) | 0.77 |
| **Cavity on CXR**  No  Yes | **n=93**  46 (49.5)  47 (50.5) | **n=156**  86 (55.1)  70 (44.9) | 0.38 | 33 (61.3)  20 (38.7) | 22 (46.2)  26 (53.8) | 0.20 |
| The dependent outcome for Crude RR: successful outcome  BMI: Body Mass Index; DM: Diabetes Mellitus; CKD: Chronic Kidney Disease; TB: Tuberculosis; CXR: Chest x-ray; RR: Relative Risk; CI: Confidence Interval. Categorical variables were presented as number (%)  * Statistically significant (p<0.05) | | | | | | |

**S2 Table. Analysis of Excluded Patients.**
