# Supplementary material for: Factors associated with treatment outcome of MDR/RR-TB patients treated with shorter injectable based regimen in West Java Indonesia
Source: PLoS One. 2022 Jan 28;17(1):e0263304. doi: 10.1371/journal.pone.0263304 (PMC8797248; doi:10.1371/journal.pone.0263304)
Supplement: S2 File — (PDF) [file pone.0263304.s003.pdf]

UNIVERSITAS PADJADJARAN

DIRECTORATE OF RESEARCH AND COMMUNITY ENGAGEMENT

PROOFREADING CERTIFICATE

This document certifies that the manuscript listed below has been proofread for proper English language, grammar, punctuation, spelling by one or more of the qualified proofreaders.

Manuscript title:

Factors Associated with Successful Outcome of Multi Drug Resistant TB Patients Treated with Shorter Regimen in Jawa Barat, Indonesia

Author(s):

Arto Yuwono Soeroto, Raden Desy Nurhayati, Aga Purwiga, Bony Wiem Lestari, Chica Pratiwi, Prayudi Santoso, Iceu Dimas Kulsum, Hendarsyah Suryadinata, Ferdy Ferdian

Date issued:

June 22nd, 2021

Certificate Verification Key:

DRPM/PR/0637.07/2021

Neither the research content nor the authors' intentions were altered in any way during the proofreading process. Documents receiving this certification should be English-ready for publication, however, the author has the ability to accept or reject our suggestions and changes. Should you have any questions or concerns about this proofread document, please contact proofreading clinic at riset@unpad.ac.id

Proofreading Clinic | Copyright © 2017 DRPMI UNPAD. All rights reserved
